# When the parts are greater than the whole: how understanding mechanisms can advance implementation research

**DOI:** 10.1186/s13012-025-01427-6

**Published:** 2025-05-13

**Authors:** Elvin H. Geng, Byron J. Powell, Charles W. Goss, Cara C. Lewis, Anne E. Sales, Bo Kim

**Affiliations:** 1 Department of Medicine, Division of Infectious Diseases, Washington University in St. Louis, St. Louis, USA; 2 Center for Dissemination and Implementation in the Institute for Public Health, Washington University in St. Louis, St. Louis, USA; 3 Brown School of Social Work, Washington University in St. Louis, St. Louis, USA; 4 Institute for Informatics, Data Science and Biostatistics, Washington University in St. Louis, St. Louis, USA; 5 Kaiser Permanente Washington Health Research Institute, Seattle, WA USA; 6 Sinclair School of Nursing and Department of Family and Community Medicine, University of Missouri, Columbia, MO USA; 7 VA Ann Arbor Healthcare System, Ann Arbor, MI USA; 8 VA Boston Healthcare System and Department of Psychiatry, Harvard Medical School, Center for Healthcare Organization and Implementation Research, Boston, USA

**Keywords:** Context, Generalizability, Mechanisms, Implementation strategies

## Abstract

**Background:**

Does the importance of context in implementation imply that generalizing about the effects of strategies is ultimately limited? Conceptual approaches for generalizing in the presence of significant contextual heterogeneity could advance implementation research but require novel perspectives.

**Main body:**

Drawing from perspectives from Realist approaches, Pearl’s transportability framework and philosophy of science, this paper outlines a mechanism-based approach to generalizing about the effects of implementation strategies. We suggest that understanding mechanisms creates a conceptual bridge between the effects of a strategy and the influence of the implementation context. Using directed acyclic graphs to represent the mechanisms of strategies, we show how conceptualizing mediators of overall effects offer a basis for considering the effects of context. Hence, theorizing and testing a mechanistic understanding enriches the ways in which we can consider how context could change those effects. Such an approach allows us to understand how a strategy works within a given implementation context, determine what information from new contexts are needed to infer across contexts, and if that information is available, what those effects would be — thereby advancing generalizing in implementation research. We consider particular implementation strategies (e.g., Community Adherence Groups and practice facilitation) as examples to illustrate generalizing into different contexts.

**Conclusion:**

Mechanisms can help implementation research by simultaneously accommodating the importance of context as well as the imperative to generalize. A shift towards a mechanism-focused approach that goes beyond identifying barriers and facilitators can enhance the value of implementation research.

Contributions to the literature

- Explicating the mechanisms of implementation strategies — the sum of the pathways that mediate their effects — offers a conceptual basis for considering how contexts can influence those effects
- Given the need for inferring across implementing contexts, a mechanistic explication of implementation strategies is an important research priority for implementation research
- Mechanisms can help move the field from the well-established observation that context matters toward understanding of context that matters and how, when and where it matters

## Implementation science: generalizing and its discontents

While the science of implementation has made tremendous strides, some fundamental questions remain incompletely settled. One such question is whether the diversity of implementation contexts (e.g., varying organizational, financial and social factors) constrain or even preclude generalizing about the effects of implementation strategies (in which we study a particular group and apply findings to a larger target group). Context is widely seen as an important driver of implementation success [1–5]. As a consequence, therefore, differences across implementing contexts [6] imply that implementation strategies will have different effects in many of these different contexts. Empiric studies confirm suspected heterogeneity. For example, systematic reviews of audit and feedback [7] — a widely used strategy — find effects that range from −30% to + 300% [8, 9]. While some drivers of differences are known (e.g., the intensity of the intervention, the level of performance at baseline), context likely also plays an important role [9].

If every implementation context — defined by a particular combination of organizational, social, and human features — is truly distinctive (and not entirely measurable), how can we generalize from a particular research study about an implementation strategy to the wider world? Natural sciences, for example, claim laws that are invariant almost everywhere (e.g., laws of gravity). Interventions identified in biological sciences (e.g., medications) also have clinical effects that apply broadly: medications for treating HIV, tuberculosis, hypertension, and other disorders essentially work if used in virtually all people with those conditions, irrespective of physiological, social, economic, or other contexts [10]. Some perspectives from social and behavioral sciences — which are closer to implementation research — doubt that we can generalize from particulars to the wider world because contexts are too heterogenous. Instead, they emphasize conceptual abstraction or case-to-case transfer as alternatives [11]. Can we generalize from particulars to diverse external contexts in implementation research, and if so, how?

In this paper, we suggest that understanding *mechanisms* of implementation strategies provides a helpful approach to generalizing from a study to wider world in implementation science. We draw from recent papers about mechanisms in implementation science and also from philosophy of science [12], causal inference, policy analysis [13], and Realist evaluation [14]. We argue that generalizing about effects of a strategy tested in a particular context into diverse external contexts is possible even when effects differ in those target contexts. We show how to do so through combining an understanding of the mechanisms of a strategy (derived in part from studies with information from implementation contexts outside of those studies (into which we want to infer). This approach has implications for study design, measurements and the types of questions prioritized in the field of implementation research.

### Generalizing 1.0: external validity

**Fig. 1:**
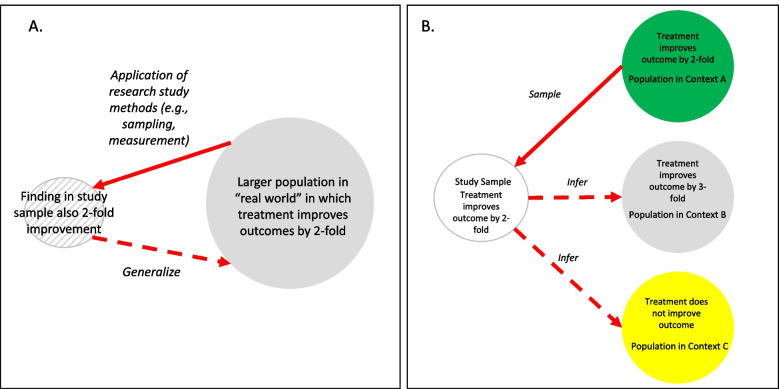
Traditional vs. Modern Generalization. A traditional approach to generalizing take effects observed in a study and seeks to apply those effects in a larger population external to the study (Panel **A**). If the underlying units of interest (whether patient, providers or health care units) is well characterized, the sampling probabilities into the study are known, typical threats to validity are adequately managed (e.g., measurement error), and the study itself does not create an artifactual environment, findings can be used to infer (with statistical uncertainty) about effects in the external population and contexts. Implementation research often assumes, in contrast, there is a meaningful diversity of contexts in the real world. This implies that the effects of any implementation strategy will differ across those contexts. Instead of identifying a single effect that applies in all contexts, the field may need to seek effects in one context in a way that enables inferring in other external contexts (Panel **B**), even when the effects will differ. We seek an approach to generalizing such that a study (Panel B) in one of the three contexts (Context A) can be used to infer about effects in other contexts such as Context B (where the strategy improves outcomes by threefold) or Context C (where the strategy has no effect). Is that possible?

Traditionally, one of the prevailing paradigms about generalizing research findings conceives of the process as taking a result observed in a study (or set of studies), and applying that result to an external “real-world” context outside of those studies [15]. This type of generalizing assumes that there is one meaningful answer in that “real-world.” Under such an assumption, finding the “real-world” answer in research depends on conducting studies in the organizational, social, economic and demographic contexts that are representative of the “real-world” (Fig. 1a). External validity can therefore be achieved through avoiding research related artifacts and rigorous research designs to manage bias. If successful, a study will therefore reasonably approximate effects in the “real-world.” The critical assumption is that there is one meaningful real-world effect provides a premise for generalizing (Fig. 1a). Pragmatic trial designs, for example, seek to ensure applicability in the real world through studying patients in care settings that are typical of real-world, interventions that are usable by typical health care delivery units, minimizing study related follow-up and other techniques [16].

Generalizing through applying a finding from a study to external real-world populations and contexts may be reasonable for biological effects of clinical interventions (e.g., medications), but may be less of a fit for implementation research. For example, clinical trials among persons living with HIV show that antiretroviral therapy with medications such as integrase inhibitors lead to biological suppression of the virus in virtually all study participants [17]. This biological effect applies virtually everywhere irrespective of social, organizational or other element of context. Likewise, the research finding that adjunctive corticosteroids in HIV-related *Pneumocystis jorvecii* pneumonia improves survival by 30%−50% [18] applies to most all patients with the PJP pneumonia. Even though there are many exceptions [19, 20] and debate about design of research to optimize this kind of generalizing continues [20], application of a clinical effects found in a study to the wider world of patients can often be valid when we are seeking to generalize biological effects [20, 21]. This simple extrapolation, however, may not work in implementation research.

### Generalizing 2.0: external validities

In implementation research, on the other hand, applying the effects of an implementation strategy found in a study (conducted in one particular context) to the external real-world is often neiher possible nor desirable. If we believe that effects of strategies (e.g., audit and feedback, practice facilitation) differ meaningfully across contexts, then research finding — even if conducted without study artifact — will be unable to directly represent effects in a meaningful range of implementing contexts. In addition, the field of implementation science is committed to finding optimal solutions in many distinctive contexts. The implied heterogeneity of the effects of implementation strategies is therefore an important feature of the field (and motivates thinking about tailoring and adaptation). Saying that any finding in a is “externally valid” is not meaningful if we accept the premise that there are many distinctive real-world contexts that influence the effects of strategies. Different effects in different contexts are not nuisances to be managed, but a feature of the reality we seek to study (Fig. 1B).

Giving up on seeking a single study effect that applies across all external contexts calls for implementation research to explore alternative approaches to generalizing from particulars to the wider world. One alternative might be to find a way to use effects found in one context to inform us about the effects in another, even if that effect differs. Instead of asking, therefore, whether a finding observed in a study conducted in Kaiser Permanente Northern California is “externally valid” or not, we would ask how we can use these finding in California to infer about effects in Michigan or Missouri — and many other places that differ in organizational, social and economic features. In short, implementation science can make generalizing tractable through modest but important adaptations to the goal of generalization.

### Mechanisms and external validities

Understanding how a strategy works — its mechanism [22] — opens the door to a kind of generalizing potentially more useful for implementation science. Drawing from approaches to understand science through explanations, we consider a mechanism to be an assembly of concepts and causal relationships that underlie an overall effect or phenomenon. One definition states that a mechanism has four basic elements: [1] an overall phenomenon or effect, (2) the parts that underlie the effect, (3) causal relationships between the parts, and (4) stable organization of the parts [23]. In this arena, “mechanisms are understood as causal systems, exhibiting a characteristic organization, with multiple causal factors that work together in a coordinated manner to produce some effect of interest.” [24].

Applied to implementation research, we see a mechanism of an implementation strategy as all the pathways through which a strategy’s effects take place. By conceptualizing the parts or components of an implementation strategy’s effects (i.e., it’s mechanism), we enable principled consideration of how a given external context could influence the effects of the implementation strategy. Each of the parts that underlie an effect (made of mediators) presents an opportunity to investigate how context might interact with that step. Mechanisms enable us to move from the truism *that context matters*, toward understanding *context that matters* by provoking theorization of (and eventually testing of) specific causal relationships. This kind of approach will allow the field to shift from seeking external validity to meaningful external *validities*.

### Using causal diagrams to explicate mechanisms

**Fig. 2:**
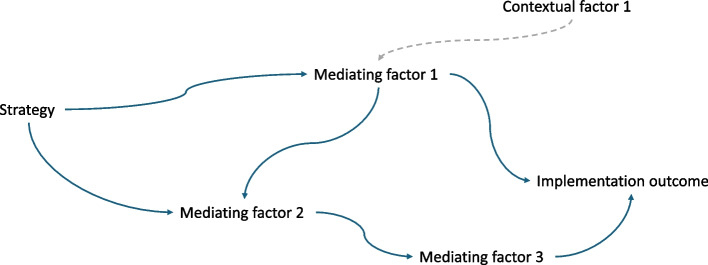
Representing mechanisms of implementation strategies. We use a diagram (using conventions of a directed acyclic graph) to illustrate the components of a mechanism. In this representation, a Strategy (S) is the implementation actions undertaken to change an implementing outcome. Mediators (M) are all the nodes or steps in a directed pathway from the Strategy to the Outcome (O) passes. Pathways (P) are all unique directed paths from the Strategy to the Outcome. For illustrative purposes, the Strategy (e.g., training) in this diagram acts through three mediators and through three different pathways. Pathway 1 is S-M1-O. Pathway 2 is S-M1-M2-M3-O. Pathway 3 is S-M2-M3-O. Note that M1 is not only a mediator of S through the S-M1-O pathway, but also a moderator of the effect of S-M2-M3-O (by also acting on M2). We consider the “mechanism” as all the mediators and pathways between S and O

In this paper, we use directed acyclic graphs (DAG’s) to represent the mechanisms of implementation strategies [25]. In these graphs — widely used in epidemiology and causal inference — arrows represent effects and nodes represent concepts (or variables in statistical parlance) (Fig. 2). Each node in a directed sequence from the strategy to the outcome is a mediator of the effects of the strategy. We refer to any directed sequence from the strategy to the outcomes as a pathway. Two arrows pointing into one node is one way to implies that the effect of one is affected by the other (a phenomenon when projected onto a numerical scale represents effect modification) [26]. When two pathways in a mechanism intersect, a mediator also acts as a moderator. We draw from the idea of “selection nodes” to represent contextual factors that differ between a source context where a study occurred and an external context [27]. These contextual factors are not on the pathway from the strategy to the effects of the strategy and therefore act only as moderators.

**Fig. 3:**
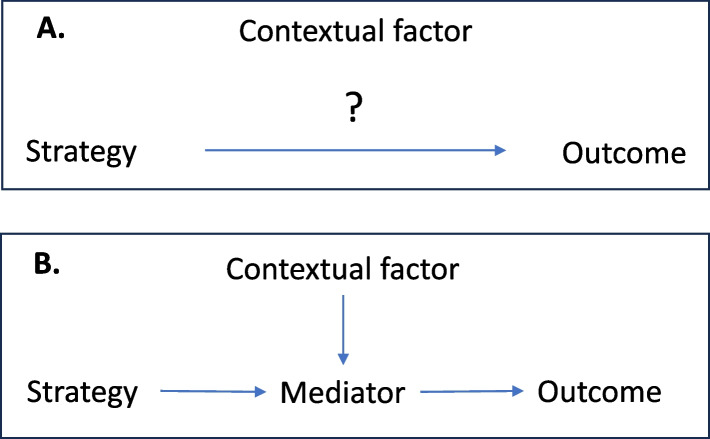
Mediators enables consideration of context. Absence of a hypothesized mechanism limits analysis of context (Panel **A**). Proposed mechanisms — in this case a single mediator for simplicity — offers a conceptual receptacle for how context might influence a strategy’s effects (Panel **B**). In the convention of directed acyclic diagrams, two arrows that point into one node implies that the effect of each arrow is influenced by the other, in this case representing how context effects the effect of the strategy on the outcome

DAG’s offer a uniquely flexible tool — but not the only one [28, 29] — for developing and testing theories about the mechanisms of implementation strategies [30]. Representing a strategy’s mechanism [22] as a DAG makes explicit how the steps in the mechanism represents a potential receptacle for contextual effects. If a strategy (S) has an effect on an outcome (O) (Fig. 3), the absence of a mediator (Z) between the strategy (S) and the outcome (O) precludes representation of how contextual factors (C) that could influence the effect of X on Z (other than through effects on O itself). When we conceptualize and represent [3] a mediator between (S) and (O), we create a receptible for the particular context’s effects on that mediator, and therefore, on the outcome as well. Causal Loop Diagrams [31] and Causal Pathways Diagrams are other graphical tools to aid thinking about contextual effects [32, 33].

Research to understand mechanisms builds on insights from Realist evaluation [34]. In the Realist perspective, effects of programs and policies are often considered instances of a theory [35]. Given the highly contextualized effects of complex programs, Realist approaches do not seek statement about whether such a program “works.” Instead, Realist evaluation describe how programs work in context [36]. Operationally, Realist evaluation uses program theory to identify specific interactions between how a program works (i.e., its mechanism) and context [37] to generate “context-mechanism-outcome” statements. Realist approaches, however, offer less emphasis on methods for using effects found in one context to infer in other, wider, external contexts [34–37]. Use of findings from one *study* context and infer in other *external* contexts (even when that effect differs) has been, on the other hand, a focus of the transportability approach pioneered by Pearl and colleagues [27, 38]. This approach “…provide[s] a formal definition of the notion of ‘transportability,’ or ‘external validity,’ as a license to transfer causal information from experimental studies to a different population.” [39] In other words, the approach uses graphical and mathematical approaches to provide a way of using knowledge found in one population to estimate effects in another [27]. The transportability approach implies that that *theorizing* about how an implementation strategy exerts it effects — the mediating pathways of the effect — are an important step in developing potentially generalizing claims. The transportability approach has not been used to date to motivate the theorization process in implementation science.

### Example community adherence groups: generalizing across contexts

We use a tangible example to illustrate how mechanisms can help with generalizing. Since 2005, rapid scale up of HIV treatment has occurred in many places of the world that previously had limited infrastructure for longitudinal outpatient care [40]. In the early phase of the global public health effort, public health agencies and governments rapidly built clinics, trained large numbers of health care workers and assembled supply chains [41]. To facilitate scale up, programs emphasized standardization. As a result, virtually all patients were given 30 days between appointments. Over time, however, it became clear that monthly visits were an untenable burden for patients over the long run [42, 43]. Newer models of care emerged, including the Community Adherence Group [44]. In this schema, six neighbors on HIV treatment would form a group and send one member each month to pick up medications for the other five members. That individual who travels on behalf of the others would also receive a bi-annual required blood tests at that visit. CAGs could be considered a type of “involve patients in implementation effort” in the Expert Recommendation for Implementing Change strategy compilation [45].

**Fig. 4:**
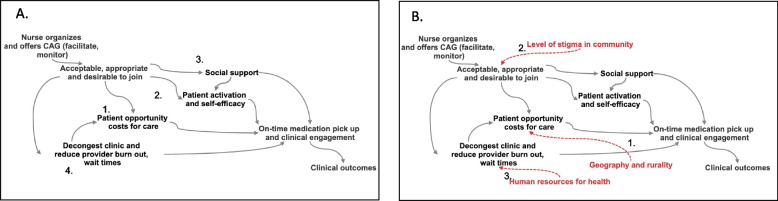
Mechanism and Context for CAG’s. A directed acyclic graph representing how a Community Adherence Group — a strategy composed of a forming a group of patients to distribute medications to each other — has effects (Panel **A**). Once offered, all effects of a CAG are mediated by acceptability and participation in the group, with subsequent steps that operate through four mediators drawn from the literature: (1) opportunity costs, (2) activation, (3) social support and (4) decongestion of clinics. Each mediator represented in this graph invites consideration of how specific contextual elements (that differ between two contexts) could influence the strategy. Each (Panel **B**) contextual effect is shown in red and includes for illustrative purposes (1) geography and rurality, (2) level of stigma and (3) human resources for health

CAGs were first shown to have effects in rural Mozambique, but would they have similar effects elsewhere? We do so by using a mechanistic explication of how CAG’s work — a descriptive theory of the strategy — based on literature to illustrate (Fig. 4) [46–48]). Published research suggests that CAG’s increase retention by reducing opportunity costs of clinical encounters (Step 1). CAG membership also was found in research to increase patient activation and accountability (Step 2 [49]) as well as enable greater peer and social support (Step 3 [48]). Finally, by decongesting clinics, CAG’s reduced size of ques and alleviated provider burden (Step 4 [47]). Some of these pathways intersect. For example, decongestion of the clinics reduces waiting times, therefore also contributing to further reduction in opportunity costs.

If CAGs have effects in rural Mozambique where they were originally studied, what effects might they have in urban Tanzania? The proposed multi-pathway mechanism of the effect of CAG’s points out contextual elements that are relevant for considering effects in the new setting (Fig. 4B).

- First, the acceptability of the CAG once offered will have important effects on the ability of the model to act through all other mechanisms (i.e., if patients refuse to join, none of the benefits will accrue). The importance of acceptability implies that differences in the level of HIV stigma across communities will play a central role in effects. Higher stigma makes disclosure of HIV status—necessary for joining a CAG— more difficult.
- Second, the rurality of the context influences the effects of CAGs that work through reducing opportunity costs. The greater the distances, the greater the benefit of membership in a CAG. Differences in distances between contexts will influence the effects of CAG’s by acting on the “opportunity cost” mediator.
- CAG’s also work through enhancing patient activation. Research suggests that differences in the level of treatment literacy, educational attainment, and self-efficacy across contexts also influence the effects of a CAG through acting on the “patient activation” node.
- Staff-to-patient ratios (which differ between rural and urban clinics) influences the effect of the CAG that operate through two pathways: reduction of opportunity costs as well as the improving the quality of the encounter. This effect, however, is likely influenced by differences in staffing and other human resources for health between two contexts . The severity of understaffing will therefore influence effects that pass through reducing patient volumes at clinic.

Each of these considerations about how contextual factors (e.g., stigma) can affect the success of offering a CAG is enabled by conceptualization of a mediator. An explanatory depiction of the effects of a strategy and the constituent parts of the effects (i.e., it’s mechanism) provides a transparent way to explore elements of context. The diagram acts as a working theory of how a CAG works and how that effect would likely vary across contexts. While in this paper we emphasize qualitative aspects, transportability also provides a mathematically grounded approach to estimate effects across different contexts.

### Example of practice facilitation: generalizing across contexts

**Fig. 5:**
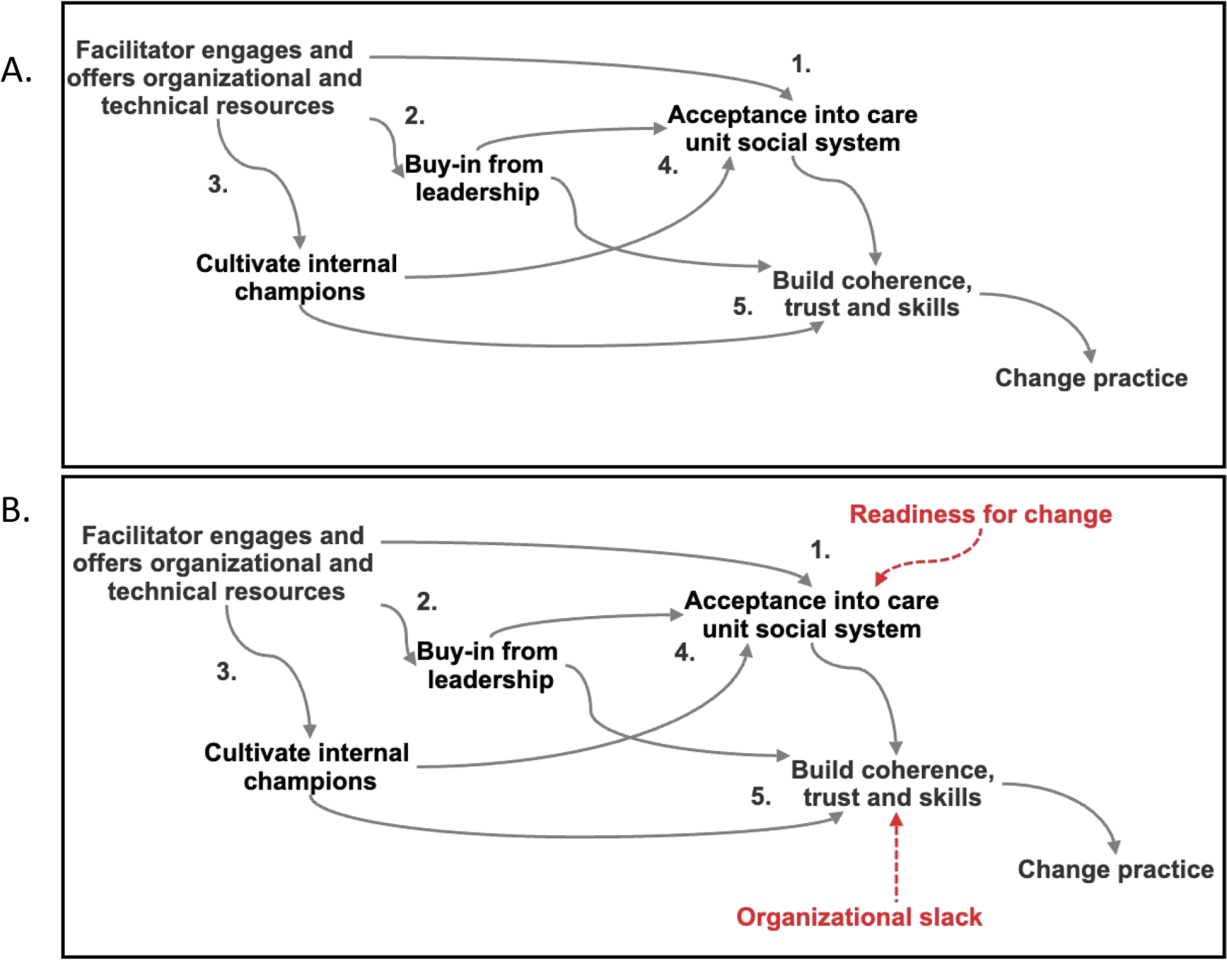
Mechanism and Context for Facilitation. Simplified mechanistic representation of healthcare facilitation for illustrative purposes. In this simplified diagram, this strategy is portrayed as a facilitator engaging with a new organizational network to provide resources and skill for change. The strategy unfolds through three key mediators: (1) acceptance into the care system, which involves gaining the healthcare system's approval and support for new practices; (2) buy-in from leadership, needed for securing broader organizational support; and (3) cultivation of internal champions who advocate for and sustain the new initiatives (Panel **A**). These elements work together to build (5) trust and coherence around the proposed activities. The diagram’s mediators invite consideration of organizational readiness for change as a contextual factor that could play a crucial role in the process by acting on acceptance (Panel **B**). The unit's ability to experiment with new activities depends on creating slack in the system—essentially, allowing resources and time for experimentation with new processes and approaches. This slack is necessary for testing and refining new ways of thinking, ensuring that the healthcare system can adapt and integrate innovative practices

Practice or healthcare facilitation [50] is often used to introduce or enhance the use of evidence-based interventions [51]. While numerous studies have demonstrated the utility or effectiveness of practice facilitation, research to understand how facilitation works [52] — mechanistically — remains an area of inquiry. A recent paper sought to use a structured process, including a Delphi process, to assemble a mechanistic representation of facilitation [51]. We use a simplified version of the mechanism from this recent paper to illustrate how a mechanistic conceptualization of healthcare facilitation enables generalizing when static effects are not probable (Fig. 5).

- This mechanism begins with an attempt by a facilitator to offer a package of socio-technical resources to a health care unit. This offer acts through acceptability of the facilitator into the health care workforce, buy in from leadership and champions (Mediators 1, 2 and 3).
- Literature points to the importance of managerial and leadership to enable facility buy-in . If this is true, then strategies for facilitation should emphasize leadership engagement not only as a means to building skills , but also in order to legitimize the facilitator within the social system of the facility.
- Leadership then acts as both a mediator of the effect as well as moderator of the effect of facilitation . Likewise, cultivating internal champions acts through building skills in their peers (as mavens of new practices) as well as on social legitimization of the facilitator.
- The diagram implies that much of the effect of facilitation are dependent on membership in the social system. This theorized mechanism also privileges (rightly or wrongly) the social aspects of facilitation and gives it a prominent place in the mechanism, as three of the five pathways operate through this mediator.
- Each of these steps then contributes to building coherence, skills, and collective action (Mediator 5), which will in turn influence practice change. We draw from Normalization Process Theory to conceptualize how facilitation changes practice through a sequence of iterative processes that transform a new practice into something that is “normal” and recedes from view [53].

The hypothesized mechanism creates an opportunity to consider how contextual factors would influence the effect (of facilitation on practice change) because it identifies the critical steps in the chain of events that constitute the effect.

- Readiness for change [54], for example, is an organizational feature that is likely widely varying and which will influence the success of the facilitator’s direct efforts as well as those mediated through leadership and champions. In other words, the right organizational environment will magnify all the pathways that pass-through acceptance.
- Likewise organizational slack [55]— surplus capacity thought to be needed for innovation — is likely to influence the effects of all efforts since all pathways pass through “coherence, participation and skills node.” In this mechanism, organizational slack therefore stands out as a distal and potentially influential contextual factor that alternations in the strength of the previous pathways will not be able to “get around.”

As in the previous example, the explanatory theory of how facilitation works, represented as a directed acyclic graph invites thinking about what and how contextual factors might be relevant.

### Implications of a mechanism-based approach for generalizing implementation strategies

A mechanisms-based approach to generalizing from a study to the wider world has important implications for the design, conduct and implications of implementation research. Some of the potential implications are subtle, but could influence the way implementation research is conducted.

#### Mechanisms can help implementation science progress as a field by enabling generalization without side-stepping context

For implementation science to mature as a field, we need to better understand the “treatment effects” of implementation strategies. Using a traditional approach to generalizing, contextual heterogeneity appears to pose a constraint to generalizing about the effects of strategies — and therefore a challenge to the maturation of the field more generally. A more nuanced approach to generalizing, based on mechanisms, can help overcome this impasse and advance the goals of the field. The field of implementation science initially sought to find strategies that would work broadly, but was confronted by the realization that diverse contexts meant invariant effects were not possible. The field then turned to research on tailoring strategies so that implementation strategies could better fit a given context — with a focus on how to find what works in a particular context. Turning our attention to the mechanisms of strategies allows knowledge of how strategies interact with contexts and therefore the conditions that enable (and conversely disable) effects. In short, mechanisms can help implementation research to expand the kinds of generalizations available to the field and develop inferences about strategies for many contexts.

#### Mechanistic thinking about implementation strategies allows separating context that matters from context that does not

Mechanisms also help the field overcome the “context trap” — the sterile truism that “context matters.” The conclusion that context matters, if it goes no further, does not help advance our understanding of how to improve implementation. implementation research has sought to categorize different types of contexts . A different approach to understanding context begins with a strategy and then using the mechanism of that strategy, asks how any of the mediators of that mechanism are influenced by context. Proceeding from mechanism to context means that we create a principled way in which research can understand, distill and measure specific elements of context. Mechanism allows us to move from the belief that “context matters” to the study of “context that matters” .

Returning to the example of the CAGs, if the hypothesized mechanism of reducing opportunity costs implies that differences in the distance across two geographies is a crucial element of context. Distance between facility and residence, however, is not necessarily relevant context for all implementation strategies. For example, studies have identified poor provider-patient communication as a reason for disengagement in public health HIV treatment services [56]. As a result, some research has sought to test communication-based implementation strategies to improve use of evidence-based HIV treatment [57]. Implementation strategies based on improving communications such as these may depend on contextual factors such as worker morale or community empowerment, but context such as distance from clinic to residence — so central for generalizing about CAG’s — may not be relevant. Mechanisms therefore act as a filter for elements of context that have effects on a particular strategy.

#### Understanding the boundary of generalities allows implementation science to generalize better, not just more

A mechanism-based approach also allows us to be clear about whether can anticipate effects of a strategy in a given context and when we cannot (i.e., because we lack the information needed to make a claim). Intuitively, we know that we often need some information or assumptions about a new context to infer about a strategy’s effects in that context. Mechanisms give us one way to translate that intuition into a method. Some clinical treatments offer illustrative examples where information about a patient or group of patients is needed to anticipate effects of a treatment with a known mechanism. Medications used to treat breast cancer through blocking the estrogen receptor (e.g., tamoxifen) have clearest efficacy when used in people with breast cancer tumors that expresses the estrogen receptor [58]. Information from the patient (i.e., whether they have a tumor with the right receptor) is needed to decide whether the treatment will work or will not work. If we do not know whether the patient’s tumor expresses the receptor, we cannot say whether effects would be positive or negative. Knowledge of tumor receptor expression separates what we can claim from what we cannot. While this is a clinical and not an implementation example, by analogy, if audit-and-feedback works through highlighting a performance gap [59], then we need to know about the credibility of data systems in a given context to anticipate effects in that particular context.

#### Trials of implementation strategies should uncover mechanisms of effects to be pragmatic 

Many champion pragmatic designs as one way to create generalizable knowledge that can be applied to “real world” settings. This approach suggests that representative study populations, use of administrative data, flexible follow up and interventions that can be implemented by typical health units will give us externally valid findings [60]. This approach relies on the assumption, however, that one effect exists which could be applicable “externally.” If, however, true effects differ across context, then pragmatic trials that remove artifacts introduced by a trial environment might be necessary but not sufficient to optimize generalizing. A mechanistic approach suggests that measuring critical mediators of the strategy are useful for generalizing. This layer of measurement may be at odds with the parsimony advocated by pragmatic designs, but offers implementation researchers an alternative, and perhaps complementary, route to generalizing. Elucidating mechanisms can allow findings to be more *exportable* (using the metaphor of transportability).

One example comes from a cluster-randomized trial in Uganda. The investigators used a multi-component training-based strategy targeting the health care workers and increased HIV treatment initiation rates [61]. Qualitative data found that the approach worked ass anticipated through changing provider knowledge and attitudes. But the strategy also had unanticipated mechanisms of effect as well: even though the interventions were directed at formal health care workers, their knowledge trickled down to lay health care workers, who then introduced the ideas into the community, which meant new patients came to clinic expecting rapid treatment initiation. This pathway in the mechanism of the rapid treatment strategy — patient demand generation — implies that the effect of training health care workers could be augmented by inclusion of lay health care workers, who can then translate new practices into the community.

#### Mechanistic thinking about implementation strategies suggests that generalizing is not the property of any one study, but rather the product of knowledge across an epistemic community

A modern view of generalizing based on mechanisms also helps relieves individual investigators and their studies of an unfair burden of being solely responsible for “generalizability.” While it is true that in implementation science, we seek to design studies to be widely applicable, we also cannot expect single studies to make scientific claims about use or usability in a range of environments. Research to advance a generalizable understanding of the effects of community-adherence groups would combine data about mechanisms in one context (e.g., reducing opportunity costs) with factors that affect opportunity costs in a variety of contexts (e.g., distance between residence and clinic). A trial of an implementation strategy (e.g., CAG’s) will offer only part of the picture, with the rest comprised of knowledge from an external target context. As another example, consider a study that finds that the ratio of providers to patients is the primary moderator of practice change. Measurement facility staffing could indicate whether the change could be taken up across the country. The information needed to generalize is not something that a study about a strategy can itself do, but that the wider research community is likely able to achieve.

### Limitations

This paper does not stem from, not speak to, all ontological traditions, particularly constructivist approaches and notions of transferability [62]. Of note, however, Realism — which we draw concepts from — is based on critiques of both positivism as well as constructivism [63]. Any argument about generalizing is likely to be highly contested even within an ontological perspective and is unlikely to satisfy all ontological positions and methodological approaches. Some have argued that epidemiological and statistical approaches to generalizing are inherently “colonial” — a view that some in implementation science seem congenial to [64]. That proposition would, however, implicitly epistemically malign a rising cadre of quantitative scientists, including statisticians, epidemiologists, and others in from formerly colonized societies Africa, Asia and Latin America who wield increasing global influence [65]. Nevertheless, unfair epistemic advantage are important areas for implementation science to grapple with [66]. Core functions have been proposed in implementation science as “things that work across contexts” and offer some insights into the issue of generalizing [67]. Considering them incontrovertible, however, would make the mistake of prematurely foreclosing on unresolved conversations that will ultimately strengthen the field. Finally, does explicating the basis of generalizing pose unreasonable demands on practitioners in day-to-day work? We believe that scientific explication can occur in tandem with practice in implementation science, just as it does in all social sciences.

## Conclusions

Understanding mechanisms of implementation strategies reveals contextual dependencies of the effects of those strategies. Theorizing about potential mechanisms can enable progress in research because they can conceptualize both key mediators as well as contextual factors that could affect those mediators. As these insights come into greater focus, the field may be able to better estimate the kinds of effects a set of practice and behavior change activities (whether training, audit and feedback, facilitation or myriad others) would have in a given set of contextual conditions. Generalizing in implementation science requires a more nuanced re-imagination of the scientific act of inferring broadly. While methodological challenges in the quantitative application of methods such as transportability and mediation [68] analysis exist, implementation science can also make use of qualitative insights [69] that combine mechanistic and contextual information to generalize. A mechanism-based approach advances our use barriers and facilitators by weaving concepts into an explanatory system. A mechanism-based approach also provides an antidote to “logic model” approaches that bin important concepts and thereby undermines important theorizing about the specific relationships. Taking on mechanisms in implementation science and their role in generalizing also allows engagement with other fields in social sciences to advance the field.

## Data Availability

Data sharing is not applicable to this article as no datasets were generated or analyzed during the current study.

## References

[1] Huebschmann AG, Leavitt IM, Glasgow RE. Making health research matter: a call to increase attention to external validity. Annu Rev Public Health. 2019;11:45–63. 10.1146/annurev-publhealth-. 10.1146/annurev-publhealth-040218-043945 30664836

[2] Tomoaia-Cotisel A, Scammon DL, Waitzman NJ, Cronholm PF, Halladay JR, Driscoll DL, et al. Context matters: the experience of 14 research teams in systematically reporting contextual factors important for practice change. Ann Fam Med. 2013;11(Suppl 1):S115. Available from: http://www.annfammed.org/content/11/Suppl_1/S115.abstract. 10.1370/afm.1549 PMC3707255 23690380

[3] Taylor SL, Dy S, Foy R, Hempel S, McDonald KM, Øvretveit J, et al. What context features might be important determinants of the effectiveness of patient safety practice interventions? BMJ Qual Safety. 2011;20(7):611. Available from: http://qualitysafety.bmj.com/content/20/7/611.abstract. 10.1136/bmjqs.2010.049379 21617166

[4] Dopson S, Fitzgerald L. The active role of context. In: Knowledge to action? Oxford University Press; 2005. p. 79–103.

[5] McCormack B, Kitson A, Harvey G, Rycroft-Malone J, Titchen A, Seers K. Getting evidence into practice: the meaning of `context’. J Adv Nurs. 2002;38(1):94–104. 10.1046/j.1365-2648.2002.02150.x. 11895535 10.1046/j.1365-2648.2002.02150.x

[6] Nilsen P, Bernhardsson S. Context matters in implementation science: a scoping review of determinant frameworks that describe contextual determinants for implementation outcomes. BMC Health Serv Res. 2019;19:1–21 BioMed Central Ltd. 30909897 10.1186/s12913-019-4015-3 PMC6432749

[7] Ivers N, Jamtvedt G, Flottorp S, Young JM, Odgaard-Jensen J, French SD, et al. Audit and feedback: effects on professional practice and healthcare outcomes. Cochrane Database of Syst Rev. 2012;2012(6):CD000259. 10.1002/14651858.CD000259.pub3. 10.1002/14651858.CD000259.pub3 PMC11338587 22696318

[8] Jamtvedt G, Young JM, Kristoffersen DT, O’Brien MA, Oxman AD. Does telling people what they have been doing change what they do? A systematic review of the effects of audit and feedback. Qual Saf Health Care. 2006;15(6):433. Available from: http://qualitysafety.bmj.com/content/15/6/433.abstract. 17142594 10.1136/qshc.2006.018549 PMC2464905

[9] Grimshaw JM, Thomas RE, Maclennan G, Fraser C, Ramsay C, Vale L, et al. Effectiveness and efficiency of guideline dissemination and implementation strategies HTA Health Technology Assessment NHS R&D HTA Programme. Health Technol Assess. 2004;8. Available from: www.hta.ac.uk/htacd.htm. 10.3310/hta8060 14960256

[10] Raffi F, Rachlis A, Brinson C, Arasteh K, Górgolas M, Brennan C, et al. Dolutegravir efficacy at 48 weeks in key subgroups of treatment-naive HIV-infected individuals in three randomized trials. AIDS. 2015;29(2):167–74. 25387312 10.1097/QAD.0000000000000519 PMC4284010

[11] Polit DF, Beck CT. Generalization in quantitative and qualitative research: Myths and strategies. Int J Nurs Stud. 2010;47(11):1451–8. 20598692 10.1016/j.ijnurstu.2010.06.004

[12] Machamer P, Darden L, Craver CF. Thinking about mechanisms. Philos Sci. 2000;67(1):1–25. Available from: http://www.jstor.org/stable/188611.

[13] Williams MJ. External validity and policy adaptation: from impact evaluation to policy design. World Bank Res Obs. 2020;35(2):158–91. 10.1093/wbro/lky010.

[14] Lemire S, Kwako A, Nielsen SB, Christie CA, Donaldson SI, Leeuw FL. What is this thing called a mechanism? Findings from a review of realist evaluations. New Dir Eval. 2020;2020(167):73–86.

[15] Shadish WR. The logic of generalization: five principles common to experiments and ethnographies. Am J Community Psychol. 1995;23(3):419–28.

[16] Loudon K, Treweek S, Sullivan F, Donnan P, Thorpe KE, Zwarenstein M. The PRECIS-2 tool: designing trials that are fit for purpose. bmj. 2015;350:h2147. 25956159 10.1136/bmj.h2147

[17] Walmsley S, Antonio A, Nathan C, Dan D, Andrea E, Felix G, et al. Dolutegravir plus Abacavir-Lamivudine for the treatment of HIV-1 infection. New England J Med. 2024;369(19):1807–18. 10.1056/NEJMoa1215541. 10.1056/NEJMoa1215541 24195548

[18] Bozzette S, Sattler F, Chui J, Wu A, Gluckstein D, Kemper C, et al. A controlled trial of early adjunctive treatment with corticosteroids for Pneumocystis carinii pneumonia in the acquired immunodeficiency syndrome. N Engl J Med. 1990;323(21):1451–7. 2233917 10.1056/NEJM199011223232104

[19] Andrews B, Muchemwa L, Kelly P, Lakhi S, Heimburger DC, Bernard GR. Simplified severe sepsis protocol: a randomized controlled trial of modified early goal–directed therapy in Zambia*. Crit Care Med. 2014;42(11). Available from: https://journals.lww.com/ccmjournal/fulltext/2014/11000/simplified_severe_sepsis_protocol__a_randomized.1.aspx. 10.1097/CCM.0000000000000541 PMC4199893 25072757

[20] Thake M, Lowry A. A systematic review of trends in the selective exclusion of older participant from randomised clinical trials. Arch Gerontol Geriatr. 2017;72:99–102. Available from: https://www.sciencedirect.com/science/article/pii/S0167494316302679. 28618323 10.1016/j.archger.2017.05.017

[21] Gottlieb SS, McCarter RJ, Vogel RA. Effect of beta-blockade on mortality among high-risk and low-risk patients after myocardial infarction. N Engl J Med. 1998;339(8):489–97. Available from: 10.1056/NEJM199808203390801. 10.1056/NEJM199808203390801 9709041

[22] Geng EH, Baumann AA, Powell BJ. Mechanism mapping to advance research on implementation strategies. PLoS Med. 2022;19(2):e1003918. 35134069 10.1371/journal.pmed.1003918 PMC8824331

[23] Malpas J. The stanford encyclopedia of philosophy. Zalta E, editor. 2012.

[24] Ross, Lauren, Woodward J. Causal Approaches to Scientific Explanation. Spring 2023. Zalta EN, Nodelman U, editors. The Stanford Encyclopedia of Philosophy . Metaphysics Research Lab, Stanford University; 2023. Available from: https://plato.stanford.edu/archives/spr2023/entries/causal-explanation-science/. Cited 2024 Nov 30.

[25] Digitale JC, Martin JN, Glymour MM. Tutorial on directed acyclic graphs. J Clin Epidemiol. 2022;1(142):264–7. 10.1016/j.jclinepi.2021.08.001 PMC8821727 34371103

[26] Van Der Weele TJ, Knol MJ. A tutorial on interaction. Epidemiol Methods. 2014;3(1):33–72.

[27] Pearl J, Bareinboim E. External validity: from do-calculus to transportability across populations. Statistical Science. 2014;29(4):579–95. 10.1214/14-STS486.

[28] Kislov R, Hyde P, McDonald R. New game, old rules? Mechanisms and consequences of legitimation in boundary spanning activities. Organ Stud. 2017;38(10):1421–44. 10.1177/0170840616679455.

[29] Kislov R, Pope C, Martin GP, Wilson PM. Harnessing the power of theorising in implementation science. Implement Sci. 2019;14:1 BioMed Central Ltd. 31823787 10.1186/s13012-019-0957-4 PMC6905028

[30] Meza RD, Moreland JC, Pullmann MD, Klasnja P, Lewis CC, Weiner BJ. Theorizing is for everybody: advancing the process of theorizing in implementation science. Front Health Serv. 2023;10:3. 10.3389/frhs.2023.1134931 PMC10012624 36926499

[31] Renmans D, Holvoet N, Criel B. No mechanism without context: strengthening the analysis of context in realist evaluations using causal loop diagramming. New Dir Eval. 2020;2020(167):101–14. 10.1002/ev.20424.

[32] Lewis CC, Klasnja P, Powell BJ, Lyon AR, Tuzzio L, Jones S, et al. From classification to causality: advancing understanding of mechanisms of change in implementation science. Front Public Health. 2018;6:136. 29868544 10.3389/fpubh.2018.00136 PMC5949843

[33] Lewis CC, Klasnja P, Lyon AR, Powell BJ, Lengnick-Hall R, Buchanan G, et al. The mechanics of implementation strategies and measures: advancing the study of implementation mechanisms. Implement Sci Commun. 2022;3(1):114. 10.1186/s43058-022-00358-3. 36273224 10.1186/s43058-022-00358-3 PMC9588220

[34] Pawson R, Tilley N. An introduction to scientific realist evaluation. In: Evaluation for the 21st century: a handbook. Thousand Oaks: SAGE Publications, Inc.; 1997. p. 405–18.

[35] Rycroft-Malone J, Burton CR, Wilkinson J, Harvey G, McCormack B, Baker R, et al. Collective action for implementation: a realist evaluation of organisational collaboration in healthcare. Implement Sci. 2016;11(1). 10.1186/s13012-016-0380-z PMC4748518 26860631

[36] Greenhalgh T, Kristjansson E, Robinson V. Realist review to understand the efficacy of school feeding programmes. BMJ. 2007;335(7625):858. 17954518 10.1136/bmj.39359.525174.AD PMC2043412

[37] Greenhalgh T, Wong G, Jagosh J, Greenhalgh J, Manzano A, Westhorp G, et al. Protocol—the RAMESES II study: developing guidance and reporting standards for realist evaluation. BMJ Open. 2015;5(8):e008567. Available from: http://bmjopen.bmj.com/content/5/8/e008567.abstract. 26238395 10.1136/bmjopen-2015-008567 PMC4538260

[38] Westreich D, Edwards JK, Lesko CR, Cole SR, Stuart EA. Target validity and the hierarchy of study designs. Am J Epidemiol. 2019;188(2):438–43. 10.1093/aje/kwy228. 30299451 10.1093/aje/kwy228 PMC6357801

[39] Bareinboim E, Pearl J. A general algorithm for deciding transportability of experimental results. J Causal Inference. 2013;1(1):107–34.

[40] Green A. A history of US engagement in the HIV/AIDS response. The Lancet. 2021;398(10315):1956–7.

[41] Mody A, Sohn AH, Iwuji C, Tan RKJ, Venter F, Geng EH. HIV epidemiology, prevention, treatment, and implementation strategies for public health. Lancet. 2023. Available from: https://www.sciencedirect.com/science/article/pii/S0140673623013818. 10.1016/S0140-6736(23)01381-8 38043552

[42] Roy M, Bolton Moore C, Sikazwe I, Holmes CB. A review of differentiated service delivery for HIV treatment: effectiveness, mechanisms, targeting, and scale. Curr HIV/AIDS Rep. 2019;16(4):324–34. 10.1007/s11904-019-00454-5. 31230342 10.1007/s11904-019-00454-5

[43] Ehrenkranz P, Grimsrud A, Rabkin M. Differentiated service delivery: navigating the path to scale. Curr Opin HIV AIDS. 2019;14(1). Available from: https://journals.lww.com/co-hivandaids/fulltext/2019/01000/differentiated_service_delivery__navigating_the.10.aspx. 10.1097/COH.0000000000000509 30394947

[44] Decroo T, Telfer B, Biot M, Maïkéré J, Dezembro S, Cumba LI, et al. Distribution of antiretroviral treatment through self-forming groups of patients in Tete Province, Mozambique. JAIDS J Acquired Immune Deficiency Syndromes. 2011;56(2):e39–44. 10.1097/QAI.0b013e3182055138 21084990

[45] Powell BJ, Waltz TJ, Chinman MJ, Damschroder LJ, Smith JL, Matthieu MM, et al. A refined compilation of implementation strategies: results from the Expert Recommendations for Implementing Change (ERIC) project. Implement Sci. 2015;10(1):21. 10.1186/s13012-015-0209-1. 25889199 10.1186/s13012-015-0209-1 PMC4328074

[46] Rasschaert F, Telfer B, Lessitala F, Decroo T, Remartinez D, Biot M, et al. A qualitative assessment of a community antiretroviral therapy group model in Tete, Mozambique. PLoS One. 2014;9(3):e91544-. 10.1371/journal.pone.0091544. 24651523 10.1371/journal.pone.0091544 PMC3961261

[47] Decroo T, Panunzi I, das Dores C, Maldonado F, Biot M, Ford N, et al. Lessons learned during down referral of antiretroviral treatment in Tete, Mozambique. J Int AIDS Soc. 2009;12(1):6. 10.1186/1758-2652-12-6. 10.1186/1758-2652-12-6 PMC2685122 19419543

[48] Mukumbang FC, Marchal B, Van Belle S, van Wyk B. Unearthing how, why, for whom and under what health system conditions the antiretroviral treatment adherence club intervention in South Africa works: a realist theory refining approach. BMC Health Serv Res. 2018;18(1):343. 10.1186/s12913-018-3150-6. 29743067 10.1186/s12913-018-3150-6 PMC5944119

[49] Rasschaert F, Telfer B, Lessitala F, Decroo T, Remartinez D, Biot M, et al. A qualitative assessment of a community antiretroviral therapy group model in Tete, Mozambique. PLoS ONE. 2014;9(3):e91544. 24651523 10.1371/journal.pone.0091544 PMC3961261

[50] Cohen D, McDaniel RR, Crabtree BF, Ruhe MC, Weyer SM, Tallia A, et al. A practice change model for quality improvement in primary care practice. J Healthc Manag. 2004;49(3). Available from: https://journals.lww.com/jhmonline/fulltext/2004/05000/a_practice_change_model_for_quality_improvement_in.5.aspx. 15190858

[51] Kilbourne AM, Geng E, Eshun-Wilson I, Sweeney S, Shelley D, Cohen DJ, et al. How does facilitation in healthcare work? Using mechanism mapping to illuminate the black box of a meta-implementation strategy. Implement Sci Commun. 2023;4(1):53. 10.1186/s43058-023-00435-1. 37194084 10.1186/s43058-023-00435-1 PMC10190070

[52] Sweeney SM, Baron A, Hall JD, Ezekiel-Herrera D, Springer R, Ward RL, et al. Effective facilitator strategies for supporting primary care practice change: a mixed methods study. Ann Family Med. 2022;20(5):414. Available from: http://www.annfammed.org/content/20/5/414.abstract. 10.1370/afm.2847 PMC9512557 36228060

[53] May CR, Mair F, Finch T, MacFarlane A, Dowrick C, Treweek S, et al. Development of a theory of implementation and integration: normalization process theory. Implement Sci. 2009;4(1):29. 10.1186/1748-5908-4-29. 19460163 10.1186/1748-5908-4-29 PMC2693517

[54] Weiner BJ. A theory of organizational readiness for change. Implement Sci. 2009;4(1):67. 10.1186/1748-5908-4-67. 19840381 10.1186/1748-5908-4-67 PMC2770024

[55] Gulati R, Nohria N. Is slack good or bad for innovation? Acad Manag J. 1996;39(5):1245–64.

[56] Zanolini A, Sikombe K, Sikazwe I, Eshun-Wilson I, Somwe P, Bolton Moore C, et al. Understanding preferences for HIV care and treatment in Zambia: Evidence from a discrete choice experiment among patients who have been lost to follow-up. PLoS Med. 2018;15(8):e1002636. 30102693 10.1371/journal.pmed.1002636 PMC6089406

[57] Maclachlan EW, Shepard-Perry MG, Ingo P, Uusiku J, Mushimba R, Simwanza R, et al. Evaluating the effectiveness of patient education and empowerment to improve patient–provider interactions in antiretroviral therapy clinics in Namibia. AIDS Care. 2016;28(5):620–7. 26695005 10.1080/09540121.2015.1124975 PMC4841015

[58] Osborne CK. Tamoxifen in the treatment of breast cancer. N Engl J Med. 1998;339(22):1609–18. 9828250 10.1056/NEJM199811263392207

[59] Foy R, Eccles M, Jamtvedt G, Young J, Grimshaw J, Baker R. What do we know about how to do audit and feedback? Pitfalls in applying evidence from a systematic review. BMC Health Serv Res. 2005;5(1):50. 16011811 10.1186/1472-6963-5-50 PMC1183206

[60] Ford I, Norrie J. Pragmatic trials. N Engl J Med. 2016;375(5):454–63. 27518663 10.1056/NEJMra1510059

[61] Amanyire G, Semitala FC, Namusobya J, Katuramu R, Kampiire L, Wallenta J, et al. Effects of a multicomponent intervention to streamline initiation of antiretroviral therapy in Africa: a stepped-wedge cluster-randomised trial. Lancet HIV. 2016;3(11):e539–48. 27658873 10.1016/S2352-3018(16)30090-X PMC5408866

[62] Slevin E, Sines D. Enhancing the truthfulness, consistency and transferability of a qualitative study: utilising a manifold of approaches. Nurse Res (through 2013). 1999;7(2):79.

[63] Cruickshank J. Positioning positivism, critical realism and social constructionism in the health sciences: a philosophical orientation. Nurs Inq. 2012;19(1):71–82. 22212371 10.1111/j.1440-1800.2011.00558.x

[64] Petteway RJ. On epidemiology as racial-capitalist (re) colonization and epistemic violence. Crit Public Health. 2023;33(1):5–12.

[65] Mukanga D, Tshimanga M, Wurapa F, Serwada D, Pariyo G, Wabwire-Mangen F, et al. The genesis and evolution of the African Field Epidemiology Network. Pan Afr Med J. 2011;10(1). PMC3266681 22359690

[66] Bradley CD, Irie WC, Geng EH. Situating implementation science (IS) in res (IS) tance: a conceptual frame toward the integration of scholarship from the black radical tradition. Front Public Health. 2024;11:1286156. 38274530 10.3389/fpubh.2023.1286156 PMC10808293

[67] Gesell SB, Bettger JP, Lawrence RH, Li J, Hoffman J, Lutz BJ, et al. Implementation of complex interventions: lessons learned from the patient-centered outcomes research institute transitional care portfolio. Med Care. 2021;59:S344–54. 34228016 10.1097/MLR.0000000000001591 PMC8263141

[68] Imai K, Keele L, Tingley D. A general approach to causal mediation analysis. Psychol Methods. 2010;15(4):309–34. 20954780 10.1037/a0020761

[69] Bonell C, Warren E, Melendez-Torres GJ. Methodological reflections on using qualitative research to explore the causal mechanisms of complex health interventions. Evaluation. 2022;28(2):166–81. 10.1177/13563890221086309.

